# Feasibility of a Digital Coaching Program for Improving Mental Well-Being and Emotional Intelligence: Pragmatic Retrospective Cohort Study

**DOI:** 10.2196/71828

**Published:** 2025-08-07

**Authors:** Nicholas Chalmer Peiper, Adam Pettitt, Bela Shah, Oliver Attwood, Ella Sivonen, Jeffrey Pfeffer

**Affiliations:** 1 Department of Epidemiology and Population Health, School of Public Health and Information Sciences, University of Louisville, 485 East Gray Street, Louisville, 40202, United States, 1 5028529453; 2 Center for Digital Mental Health, University of Oregon, Eugene, United States; 3 Meru Health, San Mateo, CA, United States; 4 Graduate School of Business, Stanford University, Stanford, United States

**Keywords:** mental health services, implementation science, epidemiology, digital health, quality of life, emotional intelligence

## Abstract

**Background:**

Within the past decade, digital coaching programs (DCPs) have emerged as an evidence-based modality to improve mental well-being and emotional intelligence (EI), although there is limited evidence in real-world contexts.

**Objective:**

This pragmatic retrospective cohort study aims to determine the preliminary effectiveness of a DCP in improving mental well-being and EI within a real-world context. We hypothesized that there would be a significant increase in mental well-being and EI.

**Methods:**

This study included 588 people who voluntarily enrolled in an 8-week, blended care DCP offered through their employers from October 2021 to August 2024. The DCP included routine check-ins and consultations with certified coaches. Participants completed the World Health Organization-Five Well-Being Index (WHO-5) at baseline and then weekly until the end of the program, as well as the Brief Emotional Intelligence Scale-10 (BEIS-10) at baseline and the end of the program. Multivariable linear mixed models examined changes in WHO-5 (biweekly) and BEIS-10 (pre-post) scores, adjusting for age, gender, program engagement, and program completion. Multivariable logistic regression models evaluated correlates of clinically meaningful improvements on the WHO-5 (ie, at least a 10-point improvement). We calculated a reliable change index (RCI) for the BEIS-10 and the proportion of participants meeting the RCI criterion from baseline to end of treatment.

**Results:**

In multivariate linear mixed models adjusting for demographics and program characteristics, we observed a significant increase in WHO-5 scores (baseline $\bar{x}$=45.6; week 8 $\bar{x}$=66.3; Cohen’s *d*=1.98; *P*<.001). Over half of the sample (55.4%) experienced a clinically meaningful improvement on the WHO-5. Multivariable logistic regression found that higher engagement was associated with an increased odds of a clinically meaningful improvement on the WHO-5 (adjusted odds ratio [aOR] 1.002, 95% CI 1.001‐1.003), while program noncompletion (aOR 0.27, 95% CI 0.15‐0.50) and higher baseline well-being (aOR 0.91, 95% CI 0.89‐0.92) were associated with reduced odds. BEIS-10 scores also significantly increased from baseline to the end of the program after adjusting for relevant correlates (baseline $\bar{x}$=37.6; week 8 $\bar{x}$=41.2; Cohen’s *d*=1.32; *P*<.001). The estimated RCI on the BEIS-10 was approximately 5, with 19.7% experiencing a meaningful improvement.

**Conclusions:**

These results demonstrate that DCPs can be a viable option for individuals looking to improve their mental well-being. Additional efforts should focus on establishing reliable change metrics for EI measures. Studies using hybrid effectiveness-implementation trial designs are now needed to further evaluate the real-world effectiveness of this program.

## Introduction

The United States is currently experiencing an unprecedented mental health crisis that was exacerbated by the acute phase of the COVID-19 pandemic [1,2]. US federal surveillance systems estimate that over 40% of adults are experiencing clinically significant levels of anxiety and depression symptoms in the past week [2]. The pervasiveness of mental health problems at the population level contributes to significant functional impairments, reduced workplace productivity, and disability [3-5], even among those experiencing serious psychological distress (SPD) who do not necessarily meet a clinical threshold for a formal diagnosis [6]. Moreover, research demonstrates that anxiety, depression, and SPD are highly comorbid with unhealthy behaviors and a variety of medical conditions [7,8]. As such, comprehensive prevention and treatment efforts are urgently needed to address population-level mental health problems.

Unfortunately, the US mental health care system has been experiencing significant structural barriers for over a decade [9]. The combination of stigma associated with mental health problems, inadequate reimbursement by health insurers, and a shortage of mental health providers has resulted in mental health being inadequately addressed [9,10]. It is estimated that only two-thirds of adults with depression receive any care each year, with only a small fraction of those receiving care getting high-quality, evidence-based treatment [11,12]. Even before the onset of the COVID-19 pandemic, long wait times for mental health care were common. This has been exacerbated by widespread shortages in the mental and behavioral health workforce [13]. Given the magnification of these structural barriers due to the pandemic, there remains an urgent need for new mental health care models.

Digital mental health interventions (DMHI) have recently emerged as a promising model of mental health care. DMHIs have been shown to address a number of structural barriers to care, including access, cost, and stigma [14,15]. Recent meta-analyses have found that DMHIs incorporating evidence-based modalities like cognitive behavioral therapy (CBT), mindfulness, and behavioral activation are as effective as traditional CBT in reducing depression and anxiety symptoms [16]. Because some DMHIs focus more on the treatment of clinically significant mental health symptoms rather than promoting mental well-being, they may not be the best fit for people experiencing SPD and other subclinical problems [17,18].

Recently, digital coaching programs (DCP) have emerged as viable interventions grounded in behavioral science principles to help people with subclinical mental health problems improve their mental well-being, reduce stress, and prevent the onset of mental disorders [19]. DCPs function to help people improve their well-being by building skills that increase emotional intelligence (EI), resilience, and self-actualization. Yet, studies evaluating DCPs and other coaching programs frequently lack an operational definition of what techniques were included in the interventions [20,21]. Moreover, information about the effectiveness of DCPs in real-world settings remains limited [22]. Given these gaps in knowledge and the importance of early intervention to prevent the onset of mental health problems, there is an urgent need for studies that investigate how DCPs may improve mental health and well-being.

This study evaluated the preliminary effectiveness of an 8-week DCP implemented in a real-world setting to improve mental well-being and increase EI. The primary hypothesis was that participation in the DCP would be associated with increased mental well-being and EI after adjusting for demographic and program-related factors. Exploratory analyses calculated reliable change scores for the EI measure and examined factors associated with meaningful improvements in EI.

## Methods

### Study Sample and Procedures

Program participants were enrolled in a DCP through a provider of employer-sponsored mental health care (Meru Health) between October 1, 2021, and August 19, 2024. Participants were offered the DCP through their employer. During this period, there were 45 employers offering the DCP to approximately 100,000 people. Interested participants could schedule an initial screening assessment through a digital platform. As part of this process, potential participants completed the Patient Health Questionnaire-2 and Generalized Anxiety Disorder-2 to report on depression and anxiety symptoms, respectively. To be eligible, all participants were required to have a mobile phone, be at least 18 years old, and have scores of less than 3 on the Patient Health Questionnaire-2 and Generalized Anxiety Disorder-2 [23,24]. Those reporting current or recent suicidality, substance use disorders, eating disorders, or psychotic disorders were ineligible for the program. After the screening assessment, eligible participants participated in an intake video call with a certified coach to develop a personalized care plan. Those not meeting program eligibility requirements were provided information about the 12-week treatment program that is designed for clinically significant levels of depression and anxiety symptoms.

### Ethical Considerations

This study was deemed exempt by the Pearl Institutional Review Board (21-MERU-114) for secondary analyses of previously collected and deidentified data from a program evaluation. As part of the DCP, participants consented to have their anonymized data used for evaluation and quality improvement purposes. We conducted a retrospective analysis with program data after the DCP took place. All data and protected health information were stored in a cloud storage environment compliant with the Health Insurance Portability and Accountability Act. All data were encrypted in transit, end-to-end, and at rest. Deidentified data from this study are not available in a public archive or for distribution to ensure privacy of the program participants.

### Program Description

The Meru Health DCP is a blended care intervention designed to improve well-being among people experiencing subclinical problems through a mobile phone app over an 8-week period, including face-to-face sessions, skills-building content, regular chat engagement, and digital workshops. The intervention is grounded in Daniel Goleman’s EI Framework, which posits that mental well-being and functioning may be increased by building competencies in 4 core domains of emotional intelligence: self-awareness, self-regulation, social awareness and empathy, and relationship management skills [25,26]. Each week focuses on building competencies in the 4 core EI domains, with the first 4 weeks focusing on individual-level skills and the latter 4 weeks focusing on relational skills (Multimedia Appendix 1). Participants receive a total of 32 learning videos, 40 CBT exercises, and 24 meditations spread evenly across the 8-week program. In rare situations, a participant will be referred to the therapist-supported program if they determine with their coach that therapy will be more appropriate.

The certified coaches provided personalized, daily chat support to encourage and motivate participants. Coaches are certified (Emotional Intelligence Coaching Certification, International Coaching Federation, National Board Certified Health & Wellness Coach, and Certified International Health Coach) and have training in EI, CBT, motivational interviewing, and mindfulness-based stress reduction. Participants were able to communicate with coaches via a chat support function and regular face-to-face sessions. After their intake session, on average, participants spent about 45 minutes per week on the app and engaging with their coach on various activities. Coaches used a secure, web-based dashboard developed by Meru Health to communicate with participants and monitor participant engagement metrics.

### Measures

The primary outcome was mental well-being, which was measured with the World Health Organization-5 Well-Being Index (WHO-5). The WHO-5 is a psychometrically validated screening instrument that assesses mental well-being in the past 2 weeks [27]. There are a total of 5 items that are scored on a 0‐5 Likert scale, with raw scores ranging from 0 to 25. A composite score ranging from 0 to 100 is calculated by multiplying the raw score by 4. Higher scores indicate better mental well-being. A score of 50 or less indicates poor mental well-being and suggests further clinical investigation. Participants completed the WHO-5 on a biweekly basis during the DCP. The secondary outcome was EI and measured with the Brief Emotional Intelligence Scale-10 (BEIS-10), which has established validity and reliability [28]. There are a total of 10 items scored on a 1‐5 Likert scale, with scores ranging from 10 to 50. The BEIS-10 captures dimensions of self-appraisal of emotions, appraisal of others’ emotions, self-regulation of emotions, regulation of others’ emotions, and use of emotions. Higher scores indicate better EI. Participants completed the BEIS-10 at baseline and week 8.

Program-related measures included engagement and completion. Engagement was defined as the total number of minutes participants used the app during the program. Completion was defined as the proportion of participants who actively engaged in the program for at least 4 weeks [29]. Demographics included age and gender identity (male and female). The version of the DCP (versions 1 and 2) was also included as a covariate to adjust for any potential effects of intervention modifications.

### Statistical Analyses

We plotted the average level of engagement during the intervention and fit a mixed-effects model using the *lme4* package to examine whether there was a significant change during the intervention [30]. A mixed-effects model was also used to explore significant changes in BEIS-10 scores pre- and post-intervention. Both models used age, gender, engagement, program completion, and program version as covariates. Cohen’s *d* was used to assess effect sizes in pre- and post-program WHO-5 and BEIS-10 scores. Effect sizes of 0.2, 0.5, and 0.8 were considered small, medium, and large effect sizes, respectively [31].

To determine clinically significant improvements in mental well-being and emotional intelligence [32], we calculated the proportion of participants experiencing at least a 10-point improvement on the WHO-5 from baseline to the end of the intervention [33]. Because there is no documented threshold for clinically significant improvement on the BEIS-10, we calculated a reliable change index (RCI) for the BEIS-10 and the proportion of participants meeting the RCI criterion from baseline to end of treatment [34]. We also fit a multivariable logistic regression model to determine the association between clinically significant improvement in mental well-being with program-related factors and demographics while controlling for baseline well-being, EI, and the program version.

All statistical analyses were conducted with RStudio (version 1.3.959). Statistical significance was defined as a 2-tailed *P* value≤ .05. Our analytical approach used intent-to-treat analyses whereby all participants who enrolled in the program were included regardless of engagement or completion [35]. Full information maximum likelihood was used to account for missing data within the mixed-effects models, and multiple imputation with chained equations was used to account for missingness in the modeling procedures for clinically significant improvements [36,37].

## Results

**Figure 1.**
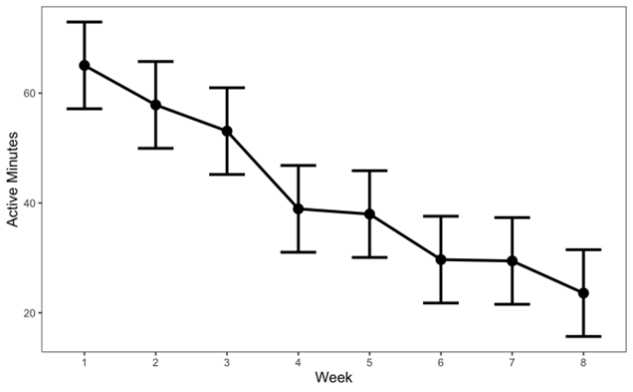
Patterns of engagement during an 8-week digital coaching program (N=588).

Overall, there were 588 people who enrolled in the Meru Health DCP between January 1, 2022, and August 19, 2024. As illustrated in Table 1, the majority of participants were female (77%), and the average age was approximately 42 (SD 11) years. The average WHO-5 score at baseline was 43 (SD 18), and the average BEIS-10 score was 37.5 (SD 4.1). A large majority of the sample (77%) participated in the DCP for at least 4 out of 8 weeks, and the total number of minutes active during the DCP was 367 (SD 343). The estimated marginal mean (EMM) number of minutes participants were active in the DCP app was 49.15 (95% CI 44.1‐54.2) during the first week, then decreased in a linear fashion to 7.67 (95% CI 2.61‐12.7) minutes at week 8 (Figure 1). No significant differences between program versions were found in the demographic characteristics and engagement.

**Table 1.** Characteristics of participants in an 8-week digital coaching program (N=588).

Characteristic	Overall	V1^a^ program (n=96)	V2 program (n=492)	*P* value^b^
Completion, n/N (%)				.049
Completer	450/588 (77)	66/96 (69)	384/492 (78)	
Dropout	138/588 (23)	30/96 (31)	108/492 (22)	
Age (years), mean (SD)	42 (11)	42 (11)	42 (11)	.70
Gender, n/N (%)				.50
Female	451/588 (77)	76/96 (79)	375/492 (76)	
Male	137/588 (23)	20/96 (21)	117/492 (24)	
Total minutes active, mean (SD)	367 (343)	212 (178)	398 (359)	<.001
Baseline WHO-5^c^, mean (SD)	43 (18)	49 (16)	42 (18)	<.001
Baseline BEIS-10^d^, mean (SD)	37.5 (4.1)	38.1 (4.1)	37.4 (4.1)	.11

a V1 and V2 refer to the 2 versions of the digital coaching program.

b Pearson chi-square test and Wilcoxon rank sum test.

c WHO-5: World Health Organization-5 Well-Being Index.

d BEIS-10: Brief Emotional Intelligence Scale-10.

### Mental Well-Being Outcomes

**Figure 2.**
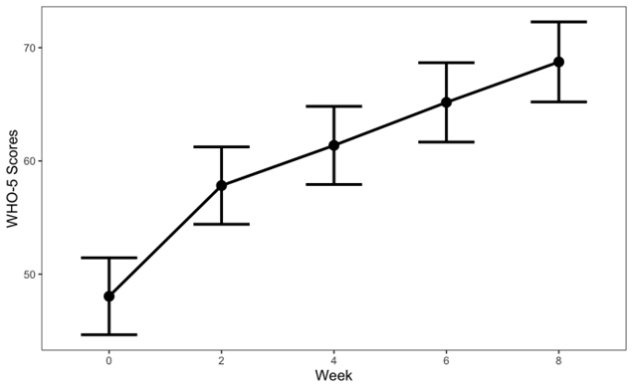
Change in WHO-5 scores during an 8-week digital coaching program (N=588). Error bands indicate 95% CIs. WHO-5 scores range from 0 to 100, with higher scores indicating better mental well-being. Scores of 50 or less indicate poor mental health requiring clinical attention. WHO-5: World Health Organization-Five Well-Being Index.

**Figure 3.**
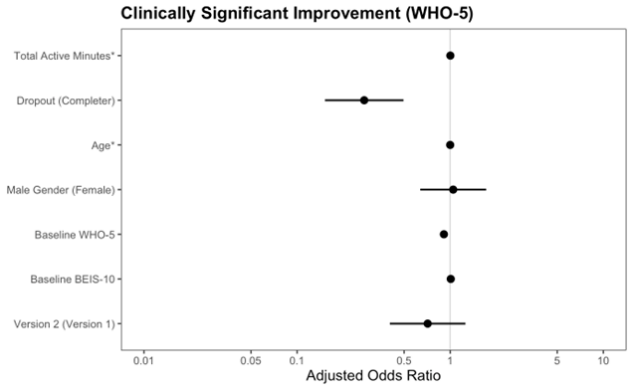
Multivariate associations with clinically significant improvement in mental well-being (N=588) Program noncompletion was defined as engaging in the program for less than 4 weeks. Reference groups are included parenthetically. Total active minutes, age, baseline WHO-5, and baseline BEIS-10 do not show visible CIs due to their extremely narrow ranges relative to other predictors. * indicates continuous variables. WHO-5: World Health Organization-Five Well-Being Index; BEIS-10: Brief Emotional Intelligence Scale-10.

We conducted mixed-effects modeling to determine WHO-5 outcomes across the coaching program. Figure 2 and Table 2 depict the results of the linear mixed-effects model to determine changes in WHO-5 scores during the DCP. At baseline, the EMM for the WHO-5 was 45.6 (95% CI 43.2‐48.0), which met the established clinical threshold of 50 or less for poor mental well-being requiring clinical attention. At the end of the program, a significant increase in the WHO-5 score was observed (EMM 66.3, 95% CI 63.6‐69.0). This represented a large improvement (Cohen’s *d*=1.98, 95% CI 1.81‐2.15) that brought participants’ average WHO-5 scores outside of the clinical threshold of 50 or less. There were 55.4% who experienced a clinically meaningful improvement on the WHO-5 (ie, at least a 10-point improvement). Figure 3 shows the results from the multivariable logistic regression model examining correlates of clinically meaningful improvements in mental well-being. Higher engagement was associated with an increased odds of a clinically meaningful improvement (adjusted odds ratio [aOR] 1.002, 95% CI 1.001‐1.003). That is, the odds of a clinically meaningful improvement increased by 0.2% for every unit increase in engagement. Program noncompletion (aOR 0.27, 95% CI 0.15‐0.50) and higher baseline well-being (aOR 0.91, 95% CI 0.89‐0.92) were associated with reduced odds of a clinically meaningful improvement.

**Table 2.** Multivariable mixed-effects model results for WHO-5^a^ scores across time among participants in an 8-week digital coaching program (N=588). Reference groups are included parenthetically. For intervention weeks, the baseline assessment served as the reference group.

Predictors	WHO-5^a^				
	Estimates	Standardized beta	95% CI	Standardized 95% CI	*P* value
Intercept	47.35	−0.45	37.94‐56.77	−0.66 to−0.24	<.001
Week 2	7.61	0.46	5.00‐10.22	0.30‐0.62	<.001
Week 4	9.03	0.55	5.98‐12.08	0.36‐0.73	<.001
Week 6	15.45	0.93	12.10‐18.80	0.73‐1.14	<.001
Week 8	17.37	1.05	13.80‐20.94	0.84‐1.27	<.001
Age	0.02	0.01	−0.20 to 0.24	−0.13 to 0.16	.87
Male gender (female)	−0.63	−0.04	−5.94 to 4.67	−0.36 to 0.28	.82
Total minutes active	0.00	0.06	−0.01 to 0.01	−0.09 to 0.22	.43
Version 2 (version 1)	0.07	0.00	−4.93‐5.07	−0.30‐0.31	.98

a WHO-5: World Health Organization-5 Well-Being Index.

### Emotional Intelligence Outcomes

We also fit a separate mixed effects model with BEIS-10 scores as the outcome to determine how these scores changed from baseline to the end of treatment. Table 3 and Multimedia Appendix 2 depict the results of the model and demonstrate that from baseline to end of treatment, there was a significant increase in BEIS-10 scores. The EMM for baseline was 37.6 (95% CI 37.0‐38.1), while at the end of treatment, the EMM was 41.2 (95% CI 40.5‐41.9). This represented a large effect size (Cohen’s *d*=1.32, 95% CI 1.14‐1.50). Finally, we found an RCI of approximately 5 for the BEIS-10. Based on these criteria, 19.7% of individuals experienced a meaningful improvement in EI during the DCP.

**Table 3.** Multivariable mixed-effects model results for BEIS-10^a^ scores across time among participants in an 8-week digital coaching program (N=588). Reference groups are included parenthetically.

Predictors	BEIS-10^a^				
	Estimates	Standardized beta	95% CI	Standardized 95% CI	*P* value
Intercept	38.92	−0.15	36.55‐41.39	−0.36 to 0.06	<.001
Week 8 (baseline)	3.80	0.94	2.81‐4.80	0.69‐to 1.18	<.001
Age	−0.02	−0.04	−0.07 to 0.04	−0.19 to 0.11	.59
Male gender (female)	−0.85	−0.21	−2.26 to 0.56	−0.56 to 0.14	.24
Total minutes active	0.00	0.02	−0.00 to 0.00	−0.15 to 0.19	.79
Version 2 (version 1)	−0.38	−0.09	−1.71 to 0.94	−0.42 to 0.23	.59

a BEIS-10: Brief Emotional Intelligence Scale-10.

## Discussion

This study investigated the preliminary effectiveness of an 8-week DCP among 588 people with subclinical mental health problems. Consistent with our primary hypothesis, we observed that participation in the DCP was associated with large increases in mental well-being as measured by the WHO-5 (Cohen’s *d*=1.98). Similarly, we found that participants who did not complete the program (less than 4 weeks) had 73% lower odds of experiencing a clinically meaningful improvement in mental well-being (ie, at least a 10-point improvement on the WHO-5) compared to program completers. Exploratory analyses also revealed large increases in EI during the DCP (Cohen’s *d*=1.32) and that nearly a fifth of participants (19.7%) experienced a meaningful improvement based upon an estimated RCI of 5 on the BEIS-10 [34].

Overall, this study demonstrates that participation in a blended care DCP was associated with improvements in mental well-being among a sample of 588 adults. The results are consistent with other research studies and support the further investigation of DCPs as viable interventions to prevent the onset of common mental health problems like depression and anxiety [38]. For example, a study evaluating a blended care cognitive behavioral program delivered via video observed large improvements in depression (Cohen’s *d*=1.08) and anxiety (Cohen’s *d*=1.33) [39], which strongly aligns with the improvements in mental well-being (Cohen’s *d*=1.98) and EI (Cohen’s *d*=1.32) found in this study. Similarly, a randomized controlled trial of 146 primary care patients found that those randomized to a self-guided DCP were more likely to experience clinically significant improvements in depression and anxiety compared to a waitlist control [40]. In this study, there were large, statistically significant increases in mental well-being during the program, although the inverse association between baseline WHO-5 scores and clinically significant improvement suggests that the program had the greatest effect on participants who started with the lowest mental well-being. Thus, our results provide additional evidence that DCPs targeting people with subclinical distress may help reduce the onset of mental disorders that cause significant disability and role impairments [4,5].

Another notable finding was the relatively higher levels of completion (77%) compared to other studies. In particular, a dose-response relationship between engagement and clinically meaningful improvements in mental well-being was observed. It is possible that the continuous involvement of trained coaches over the course of the program led to higher engagement and more positive outcomes compared to self-guided or text-based DCPs [41-43]. Direct communications and involvement of trained coaches may have facilitated a remote, continuous care approach that establishes trust with participants at program intake and fosters a therapeutic alliance over the duration of the program [44,45]. This approach may have influenced the higher rate of completion observed in this study compared to other DCPs [39,46], although additional studies are needed to better evaluate how interactions with coaches (eg, frequency of coach interactions, average amount of coach time per participant) may impact adherence and program outcomes [45].

Several limitations are acknowledged. The lack of an experimental design precludes inferences about the DCP’s effectiveness in improving mental well-being and EI compared to other forms of care. Without a control group, our results are generalizable to other observational studies that cannot fully rule out natural remission of the documented changes in well-being and EI. Because participants voluntarily agreed to participate in an employer-sponsored program, we cannot rule out motivation to improve mental health and EI as an alternative explanation for the results. While self-selection may have impacted our results, the dose-response effect we observed in a real-world context is consistent with meaningful improvements in mental well-being and EI found in experimental and observational studies [18,20,39,46]. Similarly, the modest sample size represents another limitation to the study’s external validity, although our multivariate analyses were robust and highly comparable to other studies. In addition, this DCP only captured age and gender to reduce participant burden during intake, limiting inferences about race, ethnicity, and other key demographic categories. Finally, our approach to addressing missing data raises the potential for methodological artifacts. These methods assume that data for participants with missing responses on the WHO-5 and BEIS-10 may be predicted with data from those who provided responses. Because those with poorer outcomes are less likely to respond, outcomes imputed based upon the observed characteristics of participants may include inaccurate assumptions. Nonetheless, we used an intent-to-treat framework and rigorous missing data approaches rather than performing listwise deletion and conducting completer-only analyses that can lead to selection biases and inflated effect sizes [35-37,47].

Our findings indicated that participation in an 8-week DCP was associated with large improvements in mental well-being and EI. Follow-up studies with larger samples are now needed to replicate these findings and investigate the effectiveness of the DCP in preventing common mental health disorders. Hybrid implementation-effectiveness trials are also needed to generate stronger evidence of the real-world effectiveness of DCPs [48,49]. In addition, mixed methods approaches may help elucidate mechanisms related to acceptability, satisfaction, and participant-coach interactions [50]. In sum, this study provides preliminary evidence that DCPs may be viable, evidence-based interventions for improving mental well-being at the population level.

## Supplementary material

10.2196/71828 Multimedia Appendix 1 Weekly Themes and Competencies of an 8-Week Digital Coaching Program.

10.2196/71828 Multimedia Appendix 2 Change in BEIS-10 Scores During an 8-Week Digital Coaching Program (N=588).

## Abbreviations

aOR: adjusted odds ratio
BEIS-10: Brief Emotional Intelligence Scale-10
CBT: cognitive behavioral therapy
DCP: digital coaching program
DMHI: digital mental health intervention
EI: emotional intelligence
EMM: estimated marginal mean
RCI: reliable change index
SPD: serious psychological distress
WHO-5: World Health Organization-Five Well-Being Index
