# Supplementary material for: Feasibility of a Digital Coaching Program for Improving Mental Well-Being and Emotional Intelligence: Pragmatic Retrospective Cohort Study
Source: JMIR Form Res. 2025 Aug 7;9:e71828. doi: 10.2196/71828 (PMC12330984; doi:10.2196/71828)
Supplement: Multimedia Appendix 1 [file formative-v9-e71828-s001.docx]

Supplemental Table 1. Weekly Themes and Competencies of an 8-Week Digital Coaching Program

| **Weeks** | **Themes** | **Competencies** |
| --- | --- | --- |
| 1 | Turn inwards | Self-awareness, including Emotional Awareness |
| 2 | Managing emotions | Self-regulation, Self-Compassion |
| 3 | Adaptability | Stress management and responsiveness when dealing with uncertainty and change |
| 4 | Positivity | Positive outlook of self and others |
| 5 | Improving interactions with others | Social awareness, Empathy, Active Listening |
| 6 | Building effective relationships | Positive Influence |
| 7 | Managing difficult relationships | Conflict management, Healthy Boundaries |
| 8 | Conscious relationships | Being Intentional in Relationships |
