# Supplementary figures and images for: Feasibility of a Digital Coaching Program for Improving Mental Well-Being and Emotional Intelligence: Pragmatic Retrospective Cohort Study
Source: JMIR Form Res. 2025 Aug 7;9:e71828. doi: 10.2196/71828 (PMC12330984; doi:10.2196/71828)

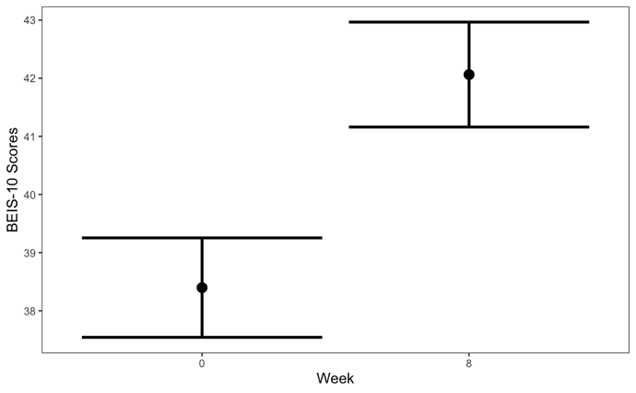

Supplement: Multimedia Appendix 2 [file formative-v9-e71828-s002.png]
